# BART Inhibits Pancreatic Cancer Cell Invasion by PKCα Inactivation through Binding to ANX7

**DOI:** 10.1371/journal.pone.0035674

**Published:** 2012-04-19

**Authors:** Keisuke Taniuchi, Kunihiko Yokotani, Toshiji Saibara

**Affiliations:** 1 Department of Pharmacology, School of Medicine, Kochi University, Nankoku, Kochi, Japan; 2 Department of Gastroenterology and Hepatology, School of Medicine, Kochi University, Nankoku, Kochi, Japan; Wayne State University School of Medicine, United States of America

## Abstract

A novel function for the binder of Arl two (BART) molecule in pancreatic cancer cells is reported. BART inhibits invasiveness of pancreatic cancer cells through binding to a Ca^2+^-dependent, phosphorylated, guanosine triphosphatase (GTPase) membrane fusion protein, annexin7 (ANX7). A tumor suppressor function for ANX7 was previously reported based on its prognostic role in human cancers and the cancer-prone mouse phenotype *ANX7*(+/−). Further investigation demonstrated that the BART–ANX7 complex is transported toward cell protrusions in migrating cells when BART supports the binding of ANX7 to the protein kinase C (PKC) isoform PKCα. Recent evidence has suggested that phosphorylation of ANX7 by PKC significantly potentiates ANX7-induced fusion of phospholipid vesicles; however, the current data suggest that the BART–ANX7 complex reduces PKCα activity. Knocking down endogenous BART and ANX7 increases activity of PKCα, and specific inhibitors of PKCα significantly abrogate invasiveness induced by BART and ANX7 knockdown. These results imply that BART contributes to regulating PKCα activity through binding to ANX7, thereby affecting the invasiveness of pancreatic cancer cells. Thus, it is possible that BART and ANX7 can distinctly regulate the downstream signaling of PKCα that is potentially relevant to cell invasion by acting as anti-invasive molecules.

## Introduction

The binder of Arl two (BART) molecule is a soluble 19-kDa protein originally purified from bovine brain and identified as a binding partner of ADP-ribosylation factor-like 2 (ARL2) [1]. The binding of BART to ARL2 is of high affinity and dependent on the binding of GTP to ARL2 [1]. Distinct functions have been inferred from findings that ARLs lack the biochemical or genetic activities characteristic of ADP ribosylation factors (ARFs), despite the 40% to 60% amino acid sequence identity between ARFs and ARLs [2]. ARL2 has been implicated as a regulator of microtubule dynamics and folding [3], but its function remains largely unknown. We previously reported that regulation of BART post-transcriptional modification *via* intracellular CD24 binding to G3BP in stress granules contributes to inhibition of invasion and metastasis of pancreatic ductal adenocarcinoma (PDAC) cells [4]. N-terminal G3BP contributes to post-transcriptional regulation of BART [5]. Further study demonstrated that BART decreases invasiveness of PDAC cells by inhibiting the ARL2-mediated decrease in the activity of the Rho GTPase protein RhoA [6]. These data suggest that BART plays a role in inhibition of PDAC invasiveness.

ANX7 is a member of the annexin family of calcium-dependent phospholipid binding proteins and codes for a Ca^2+^-activated GTPase. *ANX7*(+/−) knockout mice have Ca^2+^-dependent endocrine secretory defects [7]. ANX7 is phosphorylated by PKC, which significantly enhances binding of ANX7 to fused phospholipid vesicles in chromaffin cells [8]. Activated PKCs induce the secretion of MMP-9, lead to activation of MMP-2, downregulate TIMP-1 and TIMP-2 secretion, and increase MT1-MMP on the cell surface [9]. Thus, ANX7 may be one of the factors associated with the PKC-dependent secretion cascade. Furthermore, ANX7 is a newly described tumor suppressor gene for prostate cancer, as evidenced by loss of heterozygosity and reduced ANX7 protein expression in a large fraction of archived metastatic tumors [10]. ANX7 exhibits many biological and genetic properties of a tumor suppressor gene and is also implicated in carcinogenesis through discrete signaling pathways involving other tumor suppressors, DNA-repair and apoptosis-related genes [10], [11]. These reports have suggested that ANX7 plays several different roles involved in exocytosis, tumor suppression and carcinogenesis.

PKC is a family of serine/threonine kinases involved in the transduction of signals, including the Ras signal, for cell proliferation and differentiation [12], [13]. The PKC family consists of at least 12 isoforms with different tissue expression patterns, substrate specificities, and subcellular localizations that are related to specialized cell functions, including cell proliferation, differentiation, and apoptosis [14]. The ‘classical’ PKCs (α, β1, β2, and γ) bind phorbol esters and are Ca^2+^ dependent. The ‘novel’ PKCs (δ, ε, η, and θ) do not depend on Ca^2+^, but do bind phorbol esters. The third subfamily comprises the ‘atypical’ PKCs (ξ, ι, λ, and μ), which do not bind to either Ca^2+^ or phorbol ester [14], [15]. Constitutively activated cell surface receptors, such as the EGF receptor or the PDGF receptor [16], or Ras [17], cause hyperactivation of PKC as well as the mitogen-activated protein kinase (MAPK) cascade. Overexpression of PKCδ induces a more malignant pancreatic cancer cell phenotype *in vivo*, through modulation of cell proliferation and survival [18]. Another study demonstrated that activated PKC induces lamellipodia formation and subsequent increased migratory activity of subconjunctival fibroblasts [19]. Thus, it has been suggested that PKCs induce cell proliferation/survival, invasion and metastasis.

In this study, ANX7 was identified as a novel binding partner of BART, and BART was found to be associated with ANX7-PKC complex formation in PDAC cells. Furthermore, the current results demonstrated that knocking down BART induces cell invasion by increasing PKCα activity through the loss of ANX7-PKCα complex formation. Thus, decreased active PKCα *via* both BART and ANX7 contributes to inhibition of PDAC invasiveness.

## Results

### BART binds to ANX7 in PDAC cells

**Figure 1:**
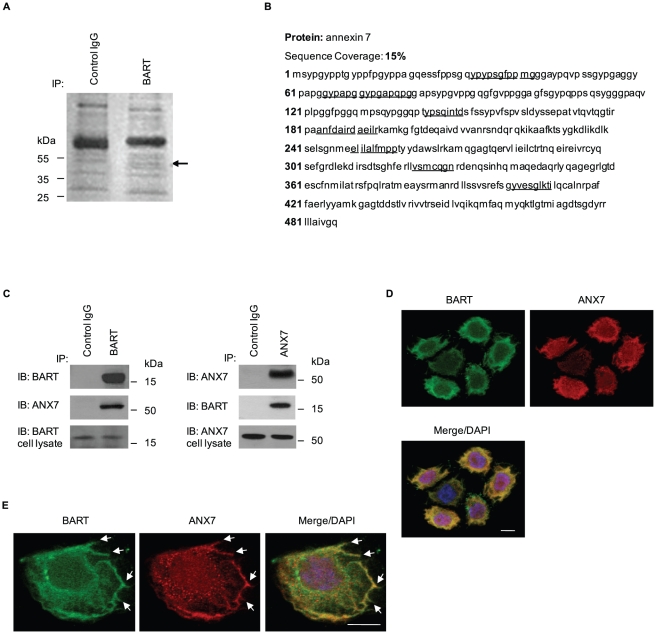
BART binds to ANX7 in lamellipodial-like protrusions. **A.** Immunoprecipitates from S2-013 cells using normal rabbit IgG (control) and anti-BART antibody were examined by silver stain analysis. Q-TOF-MS analysis investigated a prominent band in the BART immunoprecipitates (arrow). **B.** Percent coverage for ANX7 is represented by the identified peptides in the total protein sequence (accession number NM_004034). **C.** Immunoprecipitated endogenous BART or ANX7 from S2-013 were examined by Western blotting using anti-BART and anti-ANX7 antibodies. Normal rabbit or mouse IgG was used as an isotype control for BART and ANX7, respectively. **D.** Immunocytochemical staining of S2-013 cells using anti-BART (green) and anti-ANX7 (red) antibodies. Blue, DAPI staining. Bar, 10 µm. **E.** Arrows indicate that BART (green) and ANX7 (red) colocalize at lamellipodial-like protrusions of S2-013 cells. Blue, DAPI staining. Bar, 10 µm.

BART knockdown increases retroperitoneal invasion and PDAC cell metastasis to liver in an orthotopic xenograft model, as described in a previous report [4]. To investigate the mechanism by which BART suppresses invasiveness and metastasis, immunoprecipitation (IP) experiments were performed in the human PDAC cell line S2-013 using a specific antibody to BART, to detect complexes of BART with other proteins. S2-013 is a cloned subline of a PDAC cell line (SUIT-2) derived from a liver metastasis [20], and was obtained from Dr. T. Iwamura (Miyazaki Medical College, Miyazaki, Japan). Silver-stained immunoprecipitated fractions separated on SDS-PAGE gels revealed a 50-kDa band that was not seen in the isotype control immunoprecipitates (arrow in Fig. 1A). The band was excised and analyzed by Q-TOF-MS after in-gel trypsin digestion, and identified as ANX7. The peptide sequence coverage was 15% (Fig. 1B). This specific binding of ANX7 to BART was demonstrated by co-IP from S2-013 cells (Fig. 1C) and subcellular colocalization was analyzed by immunostaining of S2-013 cells (Fig. 1D). BART and ANX7 coimmunoprecipitated and were colocalized in the cytoplasm. Of note is that BART and ANX7 accumulated in lamellipodial-like protrusions that are essential for cell migration (arrows in Fig. 1E).

### ANX7 inhibits PDAC cell invasion

**Figure 2:**
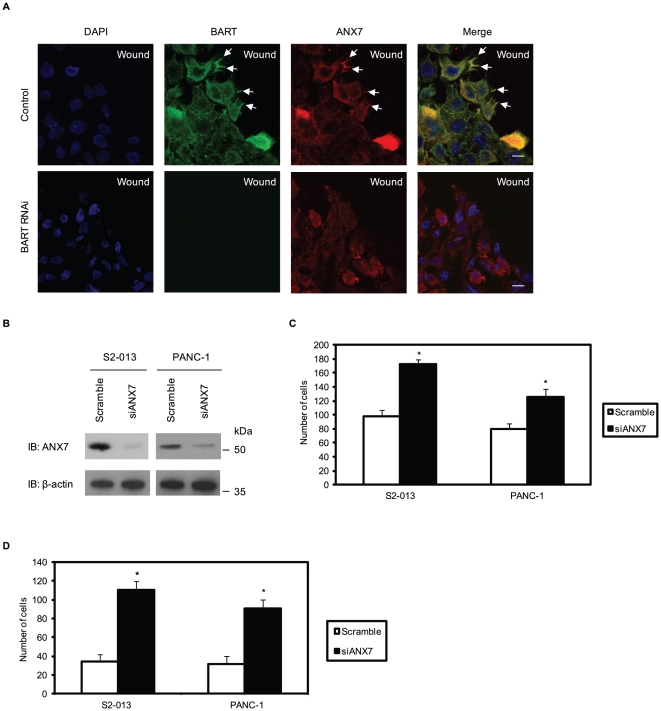
ANX7 suppresses cell motility and invasion in PDAC cells. **A.** Negative scrambled control (Scr-1) and BART RNAi (siBART-1) S2-013 cells in confluent cultures were wounded. After 4 h, the cells were immunostained using anti-BART (green) and anti-ANX7 (red) antibodies. Blue, DAPI staining. Arrows, colocalized BART and ANX7 at the leading edge of control cells. Bars, 10 µm. **B.** siRNA oligonucleotides targeting ANX7 (siANX7) and negative scrambled control were transiently transfected into S2-013 and PANC-1 cells. Western blotting validated ANX7 knockdown in both cell lines. **C.** Transwell motility assay of cells treated as in (B). Migrated cells in four fields per group were counted. Data are representative of three independent experiments. *Columns*, mean; *bars*, SD. *p<0.005 compared with control cells. **D.** Quantification of the two-chamber invasion assay of cells treated as in (B). Invaded cells in four fields per group were counted. Data are representative of three independent experiments. *Columns*, mean; *bars*, SD. *p<0.001 compared with control cells.

Previously, cell clones were generated in which BART was stably suppressed by vector-based specific short hairpin small interfering RNA (siRNA) in S2-013 cells that formerly expressed high levels of BART [4]. To determine the function of BART-ANX7 complexes, a wound-healing immunostaining assay was used to observe the localization of BART and ANX7 in polarized migrating cells (Fig. 2A). Both BART and ANX7 were recruited to the leading edges during wound healing of control S2-013 cells (arrows in Fig. 2A). Depletion of BART inhibited ANX7 accumulation at the leading edges (lower panels in Fig. 2A). Combined with the result of Fig. 1E, these results indicate that BART and ANX7 interdependently localize at the leading edges and in the lamellipodial-like protrusions associated with cell migration.

*In vitro* assays were used to examine the effects of ANX7 on cell motility and invasion. As shown by Western blot analysis, ANX7 expression was markedly reduced in S2-013 and a PDAC cell line, PANC-1, 72 h after transfection with the ANX7-targeting siRNA oligonucleotides, in contrast to cells transfected with scrambled siRNA-oligonucleotides (Fig. 2B). Suppression of ANX7 enhanced motility in transwell motility assays of S2-013 and PANC-1 as compared to control cells (Fig. 2C). In two-chamber invasion assays, ANX7 RNAi cells were significantly more invasive than the control S2-013 and PANC-1 cells (Fig. 2D). These results suggest an important role for the binding of BART and ANX7 in inhibition of cell migration.

### Binding of ANX7 and phosphorylated PKC is associated with inhibiting invasiveness of PDAC cells

**Figure 3:**
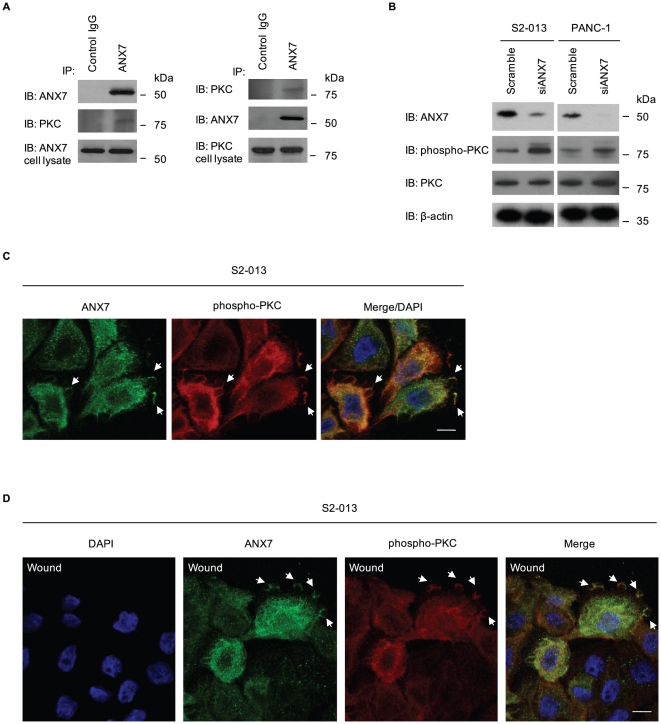
ANX7 and phosphorylated PKC are colocalized at the leading edges of migrating cells. **A.** Immunoprecipitation of endogenous ANX7 or PKC from S2-013 cells. Immunoprecipitates were examined by Western blotting using anti-ANX7 and anti-PKC antibodies. Normal mouse or rabbit IgG was used as the isotype control for ANX7 or PKC, respectively. **B.** Western blot with anti-PKC and anti-phospho-PKC antibodies showing S2-013 cells transiently transfected with siRNA for ANX7 as compared to cells transfected with scrambled control. **C.** Immunocytochemical staining in S2-013 cells, as determined with anti-ANX7 (green) and anti-phospho-PKC (red) antibodies. Blue, DAPI staining. Arrows, colocalized ANX7 and phosphorylated PKC at lamellipodial-like protrusions. Bar, 10 µm. **D.** Confluent S2-013 cells were wounded. After 4 h, the cells were immunostained using anti-ANX7 (green) and anti-phospho-PKC (red) antibodies. Blue, DAPI staining. Arrows, colocalized ANX7 and phosphorylated PKC at the leading edge. Bar, 10 µm.

Co-IP of the ANX7 and PKC complex was performed using anti-ANX7 or anti-PKC antibody (10800) reacting with the PKCα, β1, β2, δ, ε and η isoforms in S2-013 cells. Immunoblotting of the immunoprecipitates revealed that ANX7 co-immunoprecipitated with PKC (Fig. 3A). PKC expression was not particularly high, but there were significant amounts in ANX7-immunoprecipitated complexes without PKC secretagogues. The effects of knocking down ANX7 on regulating PKC activity were investigated using Western blotting using an anti-phospho-PKC antibody (9379), which detects the classical PKCs (α, β1, β2 and γ) and novel PKCs (δ, ε, η and θ) when phosphorylated at a residue homologous to Thr514 of PKCγ (Fig. 3B). ANX7 knockdown induced phosphorylation of PKC in S2-013 cells, indicating that ANX7 plays a role in decreasing phosphorylated PKC. To investigate the subcellular colocalization of ANX7 and phosphorylated PKC, S2-013 cells were immunostained. ANX7 and phosphorylated PKC were colocalized in lamellipodial-like protrusions (arrows in Fig. 3C). Interestingly, ANX7 and phosphorylated PKC were recruited and colocalized to the leading edges during wound healing of S2-013 cells (arrows in Fig. 3D), indicating that phosphorylated PKC is associated with the anti-invasive function of ANX7. Since ANX7 could function in decreasing PKC activity (Fig. 3B), ANX7-dependent inhibition of cell invasion is likely to be associated with decreased activity of the specific classical or novel PKC isoforms. Thus, further experiments for analysis of the BART-ANX7-associated inhibition of cell invasion were focused on the classical and novel PKC isoforms to which the anti-phospho-PKC antibody (9379) reacted.

### Phosphorylated PKC induces cell invasion of PDAC cells

**Figure 4:**
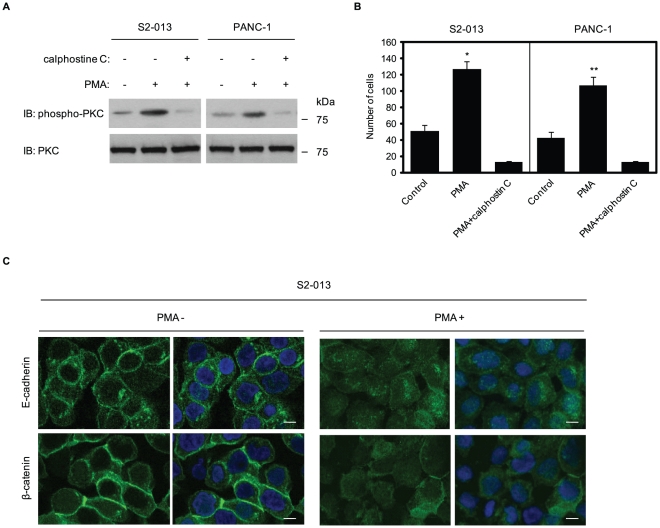
Effect of PMA on PDAC cell invasion. **A.** S2-013 and PANC-1 cells pretreated with or without calphostin C were treated with PMA, and PKC activity was assessed by Western blotting with an anti-phospho-PKC antibody. **B.** S2-013 and PANC-1 cells treated as in (A) were plated on Matrigel invasion chambers. Invaded cells in four fields per group were counted. Data are representative of three independent experiments. *Columns*, mean; *bars*, SD. *p<0.001; **p<0.003 as compared to non-treated control cells. **C.** S2-013 cells were treated with or without PMA, and immunocytochemical staining was performed using anti-E-cadherin and anti-β-catenin antibodies (green). Blue, DAPI staining; bars, 10 µm.

A PKC stimulator, phorbol 12-myristate 13-acetate (PMA) is a potent tumor promoter [21] that induces migration of subconjunctival fibroblasts [19] and glioblastoma cells [9]. PMA interacts with and activates both classical and novel PKC isoforms [21]. Therefore, the effect of PMA on cell invasion of S2-013 and PANC-1 was investigated. Immunoblotting using anti-phospho-PKC antibody (9379) revealed that treatment with PMA increased active PKCs (Fig. 4A). PMA significantly stimulated cell invasion of S2-013 and PANC-1 in *in vitro* invasion assays (Fig. 4B), indicating that PMA-sensitive PKC isoforms contribute to the invasiveness of PDAC cells. To confirm that PMA-induced invasion was dependent on active PKCs, cells were initially treated with a PKC inhibitor (calphostin C, an inhibitor of both classical and novel PKCs) and then treated with PMA. Initial treatment with the PKC inhibitor prevented the PMA-induced increase in PKC activity (Fig. 4A) and inhibited PMA-mediated cell invasion of S2-013 and PANC-1 (Fig. 4B). These results indicate that specific isoforms of classical and novel PKCs could induce PDAC cell invasion.

Cell-cell adhesion can also influence motility [22]. Upon formation of cell-cell contacts, cells reduce their migration rate and cell-surface protrusion activity, and decrease their microtubule and actin-filament dynamics [23]. To determine the effect of the classical and novel PKCs on cell-cell contact, S2-013 cells were incubated with PMA and immunofluorescence was performed using anti-E-cadherin and anti-β-catenin antibodies (Fig. 4C). PMA significantly reduced junction proteins at regions of cell-cell contact, indicating decreased peripheral localization of junction proteins, resulting in adherence junctions with decreased stability. These results suggest that PMA-sensitive PKCs play a role in decreasing stability of cell-cell contacts and, in turn, inducing cell invasion.

### BART supports the binding of ANX7 to active PKC and functions in decreasing active PKC

**Figure 5:**
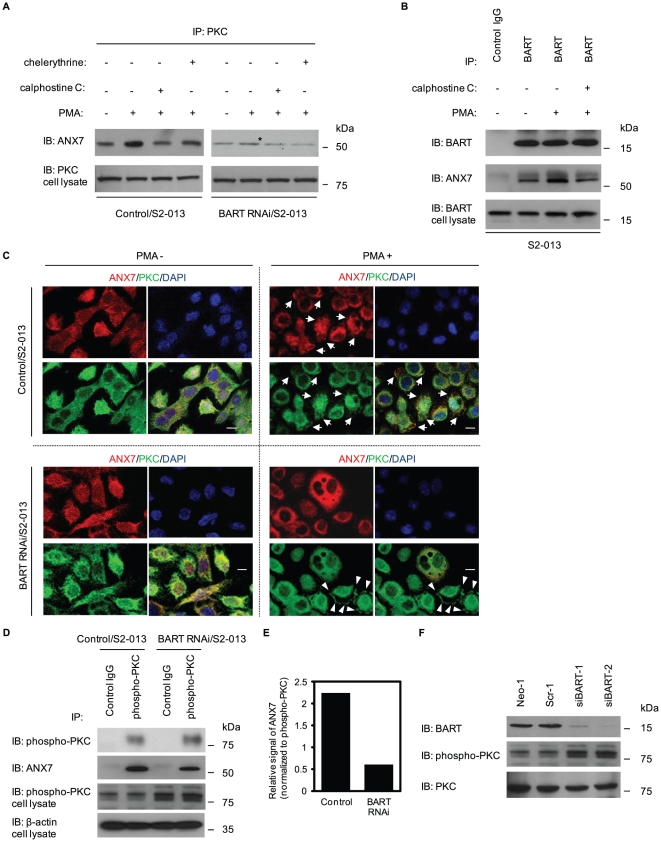
Effect of BART on regulating PKC activity through ANX7. **A.** Immunoprecipitation of PKC from control and BART RNAi S2-013 cells stimulated by PMA with or without pretreatment of calphostin C or chelerythrine chloride was examined by Western blotting using anti-ANX7 and anti-PKC antibodies. *, co-immunoprecipitated ANX7 with PKC in BART RNAi S2-013 cells. **B.** Immunoprecipitation of BART from S2-013 cells treated as in (A) was examined by Western blotting using anti-ANX7 and anti-BART antibodies. **C.** Immunocytochemical staining of control (upper panels) and BART RNAi (lower panels) S2-013 cells stimulated by PMA using anti-PKC (green) and anti-ANX7 (red) antibodies. Blue, DAPI staining. Arrows, ANX7 colocalized with PMA-sensitive PKCs in control cells; arrowheads, PMA-sensitive PKCs not colocalized with ANX7 in BART RNAi cells. Bars, 10 µm. **D.** Immunoprecipitation of phosphorylated PKC from control and BART RNAi S2-013 cells was examined by Western blotting using anti-ANX7 and anti-phospho-PKC antibodies. **E.** Densitometric analysis of the results of Figure 5D. The level of ANX7 in the precipitates was assessed after normalizing ANX7 signals to phospho-PKC signals of cell lysates. **F.** Western blot with anti-BART and anti-phospho-PKC antibodies showing two S2-013 clones transfected with siRNA for BART (siBART-1 and 2) as compared to mock (Neo-1) and scrambled (Scr-1) control clones.

To determine the effect of the BART-ANX7 complexes on regulating activity of PKC, the effects of BART knockdown on ANX7 affinity for constitutively activated PKCs by treatment with PMA were investigated (Fig. 5A). PMA stimulation caused significant increases in the amount of ANX7-PKC complexes in control cells, while there were no differences in BART RNAi S2-013 cells. Furthermore, whether binding could be inhibited by PKC inhibitors prior to stimulation of control cells with PMA was assessed. Calphostin C and chelerythrine chloride (an inhibitor of PKCα, β, γ and δ) markedly decreased ANX7-PKC interaction in PMA-stimulated control cells (Fig. 5A). In addition, BART immunoprecipitated with increased amounts of ANX7 when S2-013 cells were treated with PMA, and preincubation with calphostin C inhibited the increase in binding (Fig. 5B). Immunocytochemical analysis was performed to examine the intracellular localization of PMA-induced ANX7-PKC complexes in control and BART RNAi S2-013 cells (Fig. 5C). ANX7 colocalized with PMA-stimulated PKCs in control cells (arrows in Fig. 5C); however, BART knockdown prevented binding of ANX7 and PMA-stimulated PKCs (arrowheads in Fig. 5C). In addition, IP experiments using anti-phosphorylated PKC antibody (9379) confirmed that knocking down BART inhibited binding of ANX7 and phosphorylated PKC (Fig. 5D, E).

BART RNAi S2-013 cells had elevated active PKC levels and unchanged steady state levels of PKCs (Fig. 5F). This result indicates that BART may be associated with decreased levels of active PKC. We hypothesize that BART regulates interactions between ANX7 and active forms of target PKCs as a scaffold molecule and/or a cargo protein of ANX7, allows ANX7 to decrease target PKC activity, and in turn, inhibits cell invasion.

### PKC activity is not directly regulated by ANX7

**Figure 6:**
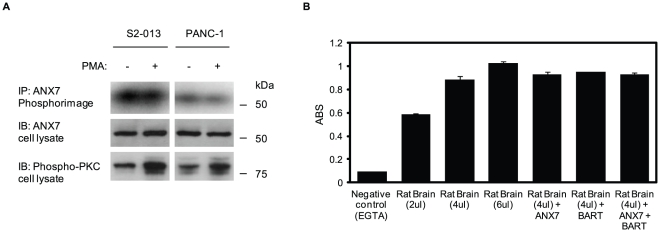
PKC phosphorylation is not directly regulated by ANX7. **A.** S2-013 and PANC-1 cells were prelabeled with [^32^P]-orthophosphoric acid and stimulated or not with PMA. ANX7 was immunoprecipitated, separated by SDS-PAGE, and analyzed by autoradiography. An immunoblot was performed on the cell lysate as a loading control. **B.** *In vitro* phosphorylation assays to investigate the effect of BART and ANX7 on PKC-phosphorylation. Purified rat brain was incubated with recombinant ANX7 with or without recombinant BART. The reaction products were analyzed by an ELISA assay. ABS on *Y*-axis means absorbance at 492 nm as reference measured with a microplate reader.

To investigate the role of PKC in regulating phosphorylation of ANX7, as previously reported in chromaffin cells [8], S2-013 and PANC-1 cells were metabolically labeled with [^32^P]-orthophosphoric acid, and then stimulated with PMA. The radioactively labeled-ANX7 was immunoprecipitated with anti-ANX7 monoclonal antibody and was analyzed by phosphor imaging (Fig. 6A). If ANX7 is a substrate of specific PKCs, the level of ANX7 phosphorylation should result in significantly increased changes in response to PMA. However, PMA stimulation did not increase the levels of ANX7 phosphorylation in either cell line. Next, *in vitro* phosphorylation assays were used to determine whether PKC activity was directly regulated by ANX7 (Fig. 6B). Purified rat brain, with a purity of >95% and containing classical and novel PKC isoforms, was incubated with recombinant ANX7 protein with or without recombinant BART protein. ANX7 did not change the activity of PKCs and adding BART protein was not associated with regulating PKC activity. These results suggest that ANX7 is not a substrate of PKC, and that ANX7 does not change phosphorylation levels of the target PKCs directly.

### PKCα is associated with BART-ANX7 complexes

**Figure 7:**
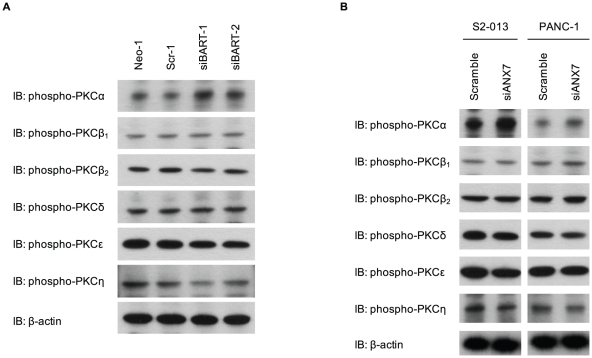
The activity of PKCα is increased by suppression of BART and ANX7. **A.** Western blot with antibodies against the classical and novel PKC isoforms showing two S2-013 clones transfected with siRNA for BART as compared to mock and scrambled control clones. **B.** siRNA oligonucleotides targeting ANX7 and negative scrambled control were transiently transfected into S2-013 and PANC-1 cells. Western blot with antibodies against the classical and novel PKC isoforms was performed.

**Figure 8:**
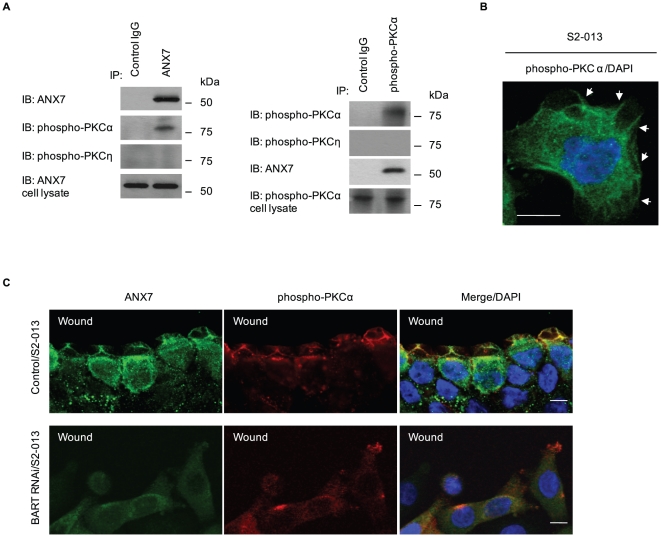
BART supports the colocalization of ANX7 and phospho-PKCα at the leading edges of migrating cells. **A.** Immunoprecipitation of ANX7 (left panels) or phospho-PKCα (right panels) from S2-013 cells was examined by Western blotting using antibodies against ANX7, phospho-PKCα and phospho-PKCη. **B.** Immunocytochemical staining in S2-013 cells, as determined with anti-phospho-PKC antibody (green). Blue, DAPI staining. Arrows, phosphorylated PKC at lamellipodial-like protrusions. Bar, 10 µm. **C.** Confluent cultures of control and BART RNAi S2-013 cells were wounded. After 4 h, the cells were immunostained using anti-ANX7 (green) and anti-phospho-PKCα (red) antibodies. Blue, DAPI staining. Bars, 10 µm.

To identify specific isoforms of PKC that bind to ANX7 in this system, a precise expression profile of classical and novel PKCs was generated by Western blotting using individual anti-phospho-PKC antibodies in BART RNAi cells derived from S2-013 (Fig. 7A). Since phosphorylation levels of target PKCs were increased in BART RNAi cells (Fig. 5F), upregulated phospho-PKCs in BART RNAi cells were selected for further analysis. Among these, PKCα was significantly activated by BART knockdown in S2-013. In addition, PKCα was abundantly phosphorylated in ANX7 RNAi cells of S2-013 and PANC-1 (Fig. 7B). Next, binding of ANX7 with phosphorylated PKCα was demonstrated by immunoprecipitation and Western blotting analysis in S2-013 cells (Fig. 8A). Phospho-PKCη was not immunoprecipitated with ANX7. To investigate the subcellular colocalization of phosphorylated PKCα, S2-013 cells were immunostained. Phosphorylated PKCα was localized in lamellipodial-like protrusions (arrows in Fig. 8B). Additionally, ANX7 and phosphorylated PKCα were recruited to the leading edges during wound healing of control S2-013 cells (upper panels in Fig. 8C). Depletion of BART did not induce accumulation of ANX7 at the leading edges and subsequent colocalization with phosphorylated PKCα (lower panels in Fig. 8C). These results indicate that PKCα is interdependently associated with BART and ANX7 in modulating PDAC cell migration.

### Specific inhibitors of PKCα inhibit increased cell migration by knockdown of BART and ANX7

**Figure 9:**
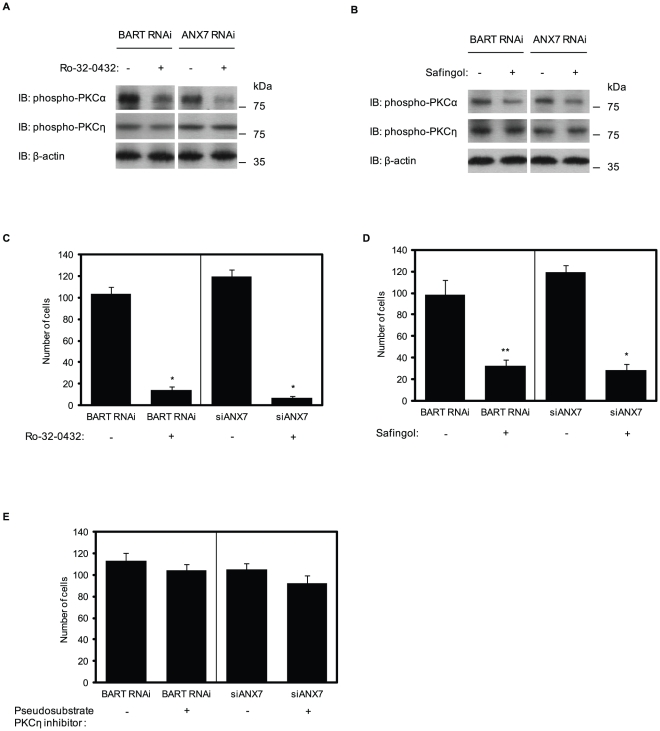
PKCα is associated with increased cell invasion by knockdown of BART and ANX7. **A.** BART RNAi S2-013 cells and S2-013 cells transiently transfected with ANX7-siRNA were pretreated with or without Ro-32-0432. The activity of PKCα and η was assessed by Western blotting. **B.** Cells shown in (A) were pretreated with or without safingol. The activity of PKCα and PKCη was assessed by Western blotting. **C.** The effect of Ro-32-0432 on cell invasion was investigated using the transwell invasion assay. Migrated cells in four fields per group were counted. Data are representative of three independent experiments. *Columns*, mean; *bars*, SD. *p<0.001 compared with non-treated cells. **D.** The effect of safingol on cell invasion was investigated using the transwell invasion assay. Data are representative of three independent experiments. *Columns*, mean; *bars*, SD. *p<0.001; **p<0.003 compared with non-treated cells. **E.** The effect of rottlerin and pseudosubstrate PKCη inhibitor on cell invasion was investigated using the transwell invasion assay. Data are representative of three independent experiments. *Columns*, mean; *bars*, SD.

To examine whether PKCα signaling is involved in increased migration by BART or ANX7 knockdown in S2-013, a PKCα/β1 inhibitor Ro-32-0432 and a specific PKCα inhibitor safingol were applied in *in vitro* invasion assays. As shown in Fig. 9A and 9B, phosphorylated PKCα was specifically decreased by Ro-32-0432 and safingol treatment, but expressions of phospho-PKCη was not changed. The greatest migration of S2-013 cells occurred after knocking down BART or ANX7; however, the increased migration was inhibited by preincubation with Ro-32-0432 (Fig. 9C) and safingol (Fig. 9D). Increased invasiveness by BART or ANX7 knockdown was not prevented by preincubation with a myristoylated pseudosubstrate PKCη inhibitor (Fig. 9E) and a PKCδ inhibitor (rottlerin; data not shown). These results suggest that activation of PKCα is required for PDAC cell migration induced by BART or ANX7 knockdown.

## Discussion

PDAC is one of the deadliest cancers due to its ability to extensively invade surrounding tissues and metastasize at an early stage [24]. Extensive local infiltration and metastasis are the main causes of death in PDAC [25]. In light of the role of BART in inhibiting PDAC cell invasion, this study was designed to identify the BART binding proteins associated with PDAC cell invasiveness. The salient features of this report are as follows:

- BART and ANX7 may function together in complex to inhibit cell migration.
- BART and ANX7 may inhibit cell invasion by decreasing active PKC at leading edges.
- Phosphorylated PKCα is responsible for the increased invasiveness of PDAC cells seen with BART or ANX7 knockdown. PKCα is interdependently associated with BART and ANX7 in modulating invasiveness of PDAC cells.

Frequent loss of ANX7 expression was observed in prostate cancer, especially in metastasis and local recurrence of hormone refractory disease [7]. Whereas null *ANX7*−/− mice die during embryogenesis, ANX7 heterozygous mice (*ANX7*+/−) develop, mature, and age normally, and more interestingly, have a cancer-prone phenotype [10]. A broad range of spontaneous tumors have been detected in *ANX7*+/− mice, including in liver, prostate, endometrium, salivary gland, and thymus [11]. In the mouse, haploinsufficiency of ANX7 expression appears to drive progression to cancer because of genomic instability through a discrete signaling pathway involving other tumor suppressor genes, DNA-repair genes, and apoptosis-related genes. Our data suggest that BART and ANX7 play a role in decreasing the phosphorylation level of PKCα in PDAC cells. *In vitro* experiments failed to demonstrate that ANX7 directly decreased activity of PKC (Fig. 6B); however, Fig. 6A shows definitively that ANX7 was not a substrate for PKC in PDAC cells. Although the mechanism by which ANX7 regulates PKC activity remains unknown, it is notable that BART and ANX7 could function together to decrease the activity of the PKCα isoform and, in turn, suppress invasiveness of PDAC cells. This study enabled evaluation of a cellular signaling pathway regulated by the *ANX7* tumor suppressor gene in PDAC. Further studies are needed to determine which molecules directly suppress PKCα, and to identify precise substrates of PKCα, in order to understand the mechanisms involved in BART-ANX7-mediated inhibition of cell invasion.

The role of PKCα in PDAC cell migration has not been extensively studied. Several lines of evidence indicate that PKCα plays critical roles in cell proliferation/migration/invasion, including human poorly differentiated hepatic cancer [26], endometrial cancer [27] and gastric cancer [28]. Cell signaling pathways involving the PKC family are initiated by binding of a ligand, such as a growth factor, to its respective cell surface receptor, which triggers the breakdown of phospholipids by phospholipases C and D and production of diacylglycerol (DAG). DAG binds to and activates most PKC isoforms, which then translocate to specific subcellular compartments that vary depending on the PKC isoform and cell type. Ultimately, MAPKs, including the extracellular-signal-regulated protein kinase (ERK), c-jun N-terminal kinase (JNK), and p38MAPK, play a crucial role in cell migration mediated by PMA-activated PKCs [19], [29]. That BART and ANX7 abrogate phosphorylation of ERK in PDAC cells has been demonstrated (data not shown), suggesting that PKCα may be associated with modulating the ERK activity possibly associated with BART-ANX7-related inhibition of invasiveness. Furthermore, it is possible that PKCα-induced exocytosis modulates cell migration. Thus, future studies of PKCα are warranted to further characterize its exocitic function and elucidate its potential contribution to cell migration.

In summary, the findings presented in this study are supportive of the pivotal roles of BART in the coordinated regulation of PKCα activity *via* binding with ANX7. The functional significance of the association of BART, ANX7 and PKCα in modulating invasiveness of PDAC cells was established. It is possible that BART and ANX7 can distinctly regulate the downstream signaling of PKCα that is potentially relevant to cell invasion by acting as anti-invasive molecules. Further studies to investigate precise mechanisms of action are warranted.

## Materials and Methods

### Reagents and antibodies

PMA and PKC inhibitors (calphostin C, chelerythrine chloride, Ro-32-0432, safingol, rottlerin and a myristoylated pseudosubstrate PKCη inhibitor) were purchased from Calbiochem (San Diego, CA). PMA and the PKC inhibitors were prepared as 10 mM stock solutions in dimethyl sulfoxide or distilled water.

Rabbit anti-BART antibody (10090-2-AP) was purchased from ProteinTech (Chicago, IL). Monoclonal antibodies against ANX7 (610669), Rac1 (610650) and β-catenin (610154) were obtained from BD Transduction Laboratory (Palo Alto, CA). Polyclonal antibodies against pan-PKC (sc-10800), PKCα (sc-208), PKCβ1 (sc-209), PKCβ2 (sc-210), PKCδ (sc-213), PKCε (sc-214) and PKCη (sc-215) were obtained from Santa Cruz Biotechnology (Santa Cruz, CA). The rabbit anti-phospho-pan-PKC antibody (9379) was purchased from Cell Signaling (Grand Island, NY), and an anti-phospho-PKCα antibody (ab23513) was obtained from Abcam (Cambridge, MA). Polyclonal antibodies against phospho-PKCβ1 (sc-101776), -PKCδ (sc-101777), and -PKCε (sc-12355) were purchased from Santa Cruz Biotechnology, and polyclonal antibodies against phospho-PKCβ2 (07-873) and -PKCη (07-877) were obtained from Millipore (Billerica, MA).

### Cell culture

The human PDAC cell line S2-013, a subline of SUIT-2, was obtained from Dr. T. Iwamura [20]. The human PDAC cell line PANC-1 was obtained from ATCC. Cells were grown in Dulbecco's modified Eagle's medium (DMEM; Gibco-BRL, Carlsbad, CA) supplemented with 10% heat-inactivated fetal calf serum (FCS) at 37°C in a 5% CO_2_, humid atmosphere.

### Immunoprecipitation and mass spectrometric analysis of BART

S2-013 cells were lysed in lysis buffer [20 mM HEPES (pH 7.4), 100 mM KCl, 5 mM MgCl_2_, 0.5% Triton X-100, and protease inhibitor cocktail tablets (Roche, Penzberg, Germany)]. Equal amounts of S2-013 cell lysates were incubated with 2 µg of anti-BART antibody or normal rabbit IgG (isotype control) and protein G Sepharose. Co-immunoprecipitated proteins were separated on a 4% to 20% gradient SDS-PAGE and then silver stained. Bands precipitated by the anti-BART antibody were excised, digested with trypsin and analyzed using a Q-TOF Ultima tandem mass spectrometer (Waters, Milford, MA) with electrospray ionization. Database searches of the acquired MS/MS spectra were performed using MASCOT v1.9.0 (Matrix Science, Boston, MA).

### In vivo binding of BART with ANX7

S2-013 cells were lysed with lysis buffer and immunoprecipitated with 2 µg of anti-BART or anti-ANX7 antibody. To examine the interaction of endogenous BART with ANX7, immune complexes were analyzed by Western blotting with anti-BART and anti-ANX7 antibodies.

### Confocal immunofluorescence microscopy

Cells were fixed with 4% paraformaldehyde, permeabilized with 0.1% Triton X-100, covered with blocking solution (3% BSA/PBS), and then incubated with the primary antibody for 1 h. Alexa 488 and Alexa 594-conjugated secondary antibodies (Molecular Probes, Carlsbad, CA) were used. Each specimen was visualized using a Zeiss LSM 510 META microscope (Carl Zeiss, Gottingen, Germany).

### siRNA-expressing constructs and the generation of stable cell lines

The methods used were as previously reported [4]. We used a pSUPERgfp vector (OligoEngine, Seattle, WA) for expression of siRNA. The target sequences for the scrambled negative control and for BART were 5′-TTCTCCGAACGTGTCACGT-3′ and 5′-CATGGCAGCCTTCACCACA-3′, respectively. S2-013 and PANC-1 cells were transfected with either empty Neo-pSUPERgfp, a scrambled oligo-pSUPERgfp negative control, or a plasmid designed to express siRNA to BART, using FuGENE6 (Roche), according to the manufacturer's instructions. Cells were selected in medium containing 500 µg/mL of geneticin to generate stable pSUPERgfp cell lines. Western blotting was performed to analyze the protein levels of the single clones.

### siRNA treatment

RNAi targeting ANX7 and scrambled negative control siRNA oligonucleotides were purchased from Santa Cruz Biotechnology (29690 and 37007). For assays to examine the effect of siRNAs on ANX7 expression, S2-013 and PANC-1 cells that express ANX7 were plated in six-well plates. After 20 h, the cells were transfected with 80 pmols of siRNA in siRNA transfection reagent (Santa Cruz), following the manufacturer's instructions.

### Transwell motility assay

Cells (3.0×10^4^) were plated in the upper chamber of BD BioCoat Control Culture Inserts (24-well plates, 8 µm pore size; Becton Dickinson, San Jose, CA). Serum-free culture medium was added to the upper chamber, and medium containing 5% FCS was added to the lower chamber. Cells were incubated on the membranes for 12 h, and motility was quantified.

### Matrigel invasion assay

The two-chamber invasion assay was used to assess cell invasion (24-well plates, 8 µm pore size, membrane coated with a layer of Matrigel extracellular matrix proteins; Becton Dickinson). Cells (4.0×10^4^) were seeded in serum-free medium into the upper chamber and allowed to invade toward 5% FCS (the chemoattractant) in the lower chamber. After 20 h incubation, the number of invading cells at the bottom of the membrane was estimated under microscopic observation by counting three independent visual fields.

BART or ANX7 RNAi S2-013 cells were preincubated for 30 min in serum-free medium containing 250 nM Ro-32-0432, 10 mM safingol, 5.0 µM rottlerin, or 5.0 µM myristoylated PKCη pseudosubstrate inhibitor. These preincubated cells were used in the two-chamber invasion assay to assess cell invasion.

### Wound healing immunostaining assay

A wound in the form of a cross was made through a confluent cell monolayer with a plastic pipette tip and cells were then allowed to polarize and migrate toward the wound. After 4 h, cells were immunostained with primary antibody and then incubated with fluorophore-conjugated secondary antibodies as described above. Each specimen was visualized with a Zeiss LSM 510 META microscope.

### Stimulation of PKC by PMA and inhibition of kinase activity by specific inhibitors

Cells were incubated in FCS-free medium for 24 h. 1 h prior to incubation with PMA, cells were preincubated at 37°C in Ca^2+^-free buffer A [25 mM Hepes (pH 7.2), 118 mM NaCl, 4.2 mM KCl, 1.2 mM MgCl_2_, 10 mM NaHCO_3_, 10 mM glucose, 0.1% bovine serum albumin] containing 50 nM calphostin C or 1.0 µM chelerythrine chloride, both specific PKC inhibitors. Next, cells were cultured for 30 min at 37°C in the presence of 100 nM PMA in buffer B (buffer A with 2.2 mM CaCl_2_ added). These cells were subjected to further analysis.

### [^32^P]-orthophosphoric acid labeling and treatment of PDAC cells with PMA and PKC inhibitors

Cells (2×10^6^/dish, 60 mm) were labeled with [^32^P]-orthophosphoric acid (1.0 mCi/ml) in phosphate-free Eagle's minimal essential medium containing 10% dialyzed FCS for 3 h at 37°C. The cells were washed once with buffer A. The cells were pretreated for 1 h at 37°C with or without the PKC inhibitors 50 nM calphostin C or 1.0 µM chelerythrine chloride in buffer A and were then stimulated to secrete by incubation for 30 min at 37°C in the presence (or absence) of 100 nM PMA in buffer B.

### Recombinant ANX7

The entire coding sequence of ANX7 cDNA was amplified by RT-PCR. The product was subsequently inserted into the pIEx-7 Ek/LIC vector (Novagen, Madison, WI) to produce a fusion protein, bearing an N-terminal 6-histidine tag. Sf9 cells (Novagen) were transiently transfected using the Insect GeneJuice Transfection Reagent (Novagen), according to the manufacturer's instructions. Transfected cells were lysed in CytoBuster Protein Extraction Reagent (Novagen) and centrifuged at 15,000× g for 15 min. The supernatant was combined with the Ni-NTA His-Bind Resin (Novagen), and bound proteins were eluted. Western blotting using an anti-ANX7 antibody was performed to identify the fractions containing ANX7. The fractions, corresponding to apparently pure proteins, were pooled, and dialyzed against storage buffer that consisted of 20 mM HEPES (pH 7.4), 20 mM KCl, and 10% glycerol. The samples were stored at −80°C.

### In vitro detection of PKC phosphorylation

A 0.05 unit (0.035 µg) of purified rat brain, with a purity of >95% and containing classical and novel PKC isoforms (Calbiochem) was incubated at 30°C for 20 min with 0.5 µmol of recombinant ANX7 with or without 0.3 µmol recombinant BART in a final volume of 30 µl of reaction buffer [25 mM Tris-HCl (pH 7.0), 5 mM β-mercaptoethanol, 3 mM MgCl_2_, 2 mM CaCl_2_, 1 mM EGTA, 0.5 mM EDTA, 0.1 mM ATP, 50 µg/ml phosphatidylserine]. To determine PKC phosphorylation, the reaction products were analyzed by an ELISA assay using PKLight Protein Kinase Assay kits (Lonza, Basel, Switzerland) according to the manufacturer's instructions.

### Statistical analysis

The significance of differences between groups was determined using the Student's *t*-test, the Mann-Whitney *U* test, or Fisher's exact test, as appropriate. Differences having *P* values <0.05 were considered statistically significant.
